# The Novel Carbapenem, JDB/PQ-1-219, Has Potent Broad Spectrum Activity against Multi-Drug Resistant *Acinetobacter baumannii*

**DOI:** 10.1021/acsinfecdis.6c00415

**Published:** 2026-06-18

**Authors:** Marta Toth, Nichole K. Stewart, Ailiena O. Maggiolo, Pojun Quan, Md Mahbub Kabir Khan, Jonathan Cox, Maidileyvis Castro Cabello, John D. Buynak, Clyde A. Smith, Sergei B. Vakulenko

**Affiliations:** † Department of Chemistry and Biochemistry, 6111 University of Notre Dame, Notre Dame 46556, Indiana, United States; ‡ 226576 Stanford Synchrotron Radiation Lightsource, Stanford University, Menlo Park 94025, California, United States; § Department of Chemistry, 2765 Southern Methodist University, Dallas 75275, Texas, United States; ∥ Department of Chemistry, Stanford University, Stanford 94305, California, United States

**Keywords:** carbapenemase, Acinetobacter baumannii, carbapenem, antibiotic, OXA-23, crystal structure

## Abstract

Carbapenem resistance in *Acinetobacter baumannii*, driven largely by class D, along with class A and class B β-lactamases, has severely compromised the utility of these last resort antibiotics. As a result, infections caused by such pathogens are characterized by extremely high mortality rates. Here we describe the antimicrobial activity of the novel C5 methyl-substituted carbapenem JDB/PQ-1–219 against multidrug resistant *A. baumannii* and the mechanism of its interaction with its major carbapenemase, OXA-23. JDB/PQ-1-219 exhibits potent antimicrobial activity against *A. baumannii* producing various carbapenemases, with MICs that are all in the clinically susceptible range. The compound has unrestricted ingress through porins and avoids egress by efflux pumps, a unique property when compared to all commercial carbapenems. Kinetic experiments demonstrated that unlike for other carbapenems, acylation of OXA-23 by JDB/PQ-1-219 is monophasic, and mass spectrometry studies showed that this results from the conversion of all enzyme into a reversible tetrahedral intermediate which gradually transitions into the stable acyl-enzyme complex. No deacylation of this complex is observed over a physiologically relevant time period, making JDB/PQ-1–219 an extremely potent inhibitor of OXA-23. Time-resolved crystallography revealed fine details of active site dynamics, leading to complete inhibition of the enzyme. Together, these studies identify JDB/PQ-1-219 as a uniquely effective novel carbapenem with clinically significant levels of activity against multidrug resistant *A. baumannii*.

## Graphical abstract

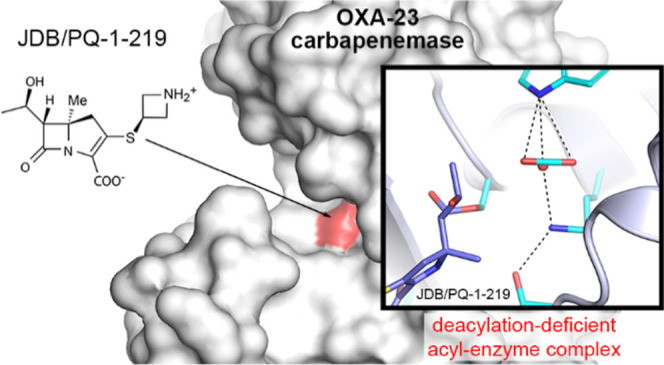

The introduction of the first antibiotics in the 1930s revolutionized the treatment of infectious diseases. However, the uncontrolled use of these drugs has driven the emergence of multidrug-resistant (MDR) pathogens, many of which have become increasingly difficult to treat, since most commonly used antibiotics are now often ineffective.^1–4^ Of particular concern are the MDR “ESKAPE” pathogens (*Enterococcus faecium* *,* *Staphylococcus aureus* *,* *Klebsiella* *pneumoniae* *,* *Acinetobacter baumannii* *,* *Pseudomonas* *aeruginosa* and *Enterobacter spp* *.*), which have become widespread, causing difficult to treat infections and creating a significant financial burden for healthcare system.^5^ Among these, the Gram-negative bacterium *A. baumannii* has emerged as one of the most notorious nosocomial pathogens. Once susceptible to most available antibiotics, it has evolved into a MDR superbug, acquiring resistance through extensive genetic exchange and genomic flexibility.^6,7^ Particularly troubling is its resistance to carbapenems, which were long considered drugs of choice for treating infections caused by this pathogen. Today, *A. baumannii* is recognized as one of the most problematic MDR pathogens,^8–10^ and is classified as a “critical” priority pathogen by the WHO^11,12^ and an “urgent threat” by the CDC.^13^

A major driver of carbapenem resistance in bacteria is the production of carbapenem-hydrolyzing β-lactamases (carbapenemases), enzymes capable of inactivating these drugs. Carbapenemases occur in three classes of β-lactamases: A and D, which use a catalytic serine, and B, which are zinc-dependent metallo-enzymes.^14^ In *A. baumannii*, carbapenem-hydrolyzing class D β-lactamases (CHDLs) are the most prevalent and fall into 19 subfamilies,^15^ seven of which, OXA-23-like, OXA-24/40-like, OXA-58-like, OXA-134-like, OXA-143-like, OXA-213-like (all acquired), and OXA-51-like (intrinsic), have recognized clinical significance. Among these, OXA-23 is by far the most widely distributed and clinically important carbapenemase in *A. baumannii*, accounting for approximately 70–80% of all acquired CHDLs.^16,17^ In addition, the class B metallo-enzyme NDM-1 has recently been also recognized as an important and growing contributor of carbapenem resistance, which further complicates therapy of multidrug resistance in this pathogen.^18^ Finally, the class A carbapenemases found in *Enterobacteriaceae* and *Pseudomonas* (the GES-type and KPC-type enzymes for example) have also been reported in *A. baumannii*.^19–21^

To combat infections caused by carbapenem-resistant pathogens, including *A. baumannii*, the continuous development of novel antibiotics and enzyme inhibitors is essential. Achieving this goal requires a detailed understanding of the enzymatic mechanism of CHDLs to inform and enable strategies that disrupt their catalytic machinery. Typically, when a carbapenem binds to a CHDL, the catalytic serine nucleophile forms a covalent and effectively irreversible acyl-enzyme complex. This complex is subsequently hydrolyzed during deacylation, releasing the inactivated antibiotic and regenerating the active enzyme. One promising strategy to improve carbapenem efficacy is to modify their canonical scaffold so that one or both steps of this catalytic process are impeded. Such modifications could transform the carbapenem from a substrate to a nonsubstrate or mechanism-based inhibitor, thereby restoring bacterial susceptibility.

We previously demonstrated that modifications to the Meropenem scaffold (Figure 1), by placing a methyl group at C5 (NA-1-157) instead of C1 (Meropenem), improved activity (relative to commercial carbapenems) against *A. baumannii* producing some CHDLs but MICs were not reduced to clinically sensitive levels.^22,23^ Here we report a novel carbapenem, JDB/PQ-1-219 (Figure 1), in which introduction of an azetidine substitution at C2 resulted in expansion of the spectrum of antimicrobial activity, allowed the compound to penetrate unimpeded through the *A. baumannii* membrane, and elevated its potency to clinically relevant levels. Our kinetic, mass spectrometry (MS), and time-resolved X-ray crystallography studies disclosed the molecular mechanism of the JDB/PQ-1-219 interaction with the major *A. baumannii* carbapenemase, OXA-23. Combined, our results demonstrate that JDB/PQ-1-219 is a potent novel carbapenem with promising potential to combat life-threatening infections caused by MDR *A. baumannii*.

**Figure 1:**
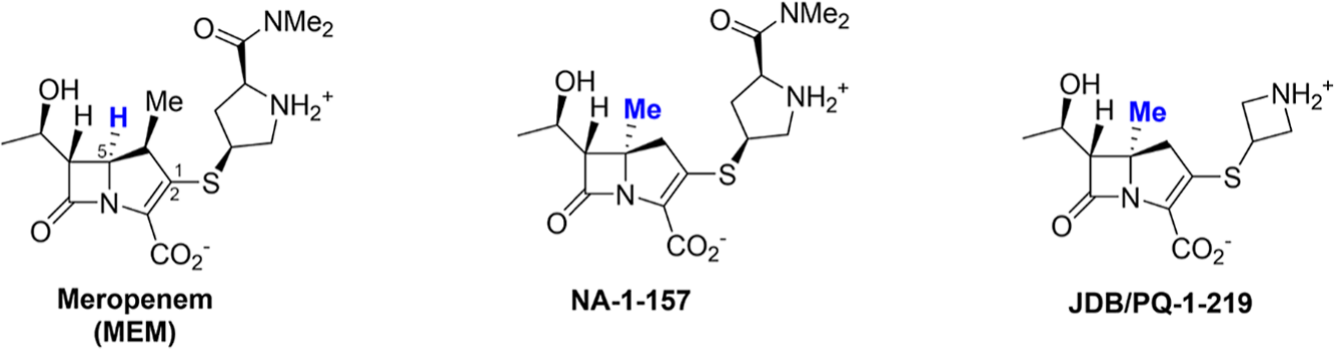
Chemical structures of carbapenems used in this study. Atom numbering is indicated on Meropenem. The C5 methyl modification present in NA-1-157 and JDB/PQ-1-219 is highlighted in blue.

## Results and Discussion

### Design and Synthesis of JDB/PQ-1-219

The detailed protocol for the synthesis of this compound from the commercially available (3*R*,4*R*)-4-acetoxy-3-[(1′*R*-*tert*-butyldimethylsilyloxy)­ethyl]-2-azetidinone **1** (XT-Anhui Puya Biological Technology CO., LTD, Anhui, China), is shown in Scheme 1 and detailed in Supporting Information Methods. Briefly, the commercially available acetate **1** was converted to 4-methylazetidin-2-one **2** using a modified Gillman reagent through an intermediate imine with the C4 stereochemistry controlled by the α-face substituent at C3. This compound was subsequently oxidized to acetate **3** using a ruthenium promoted process, again with stereochemistry of the acetate controlled by the α-face C3 substituent.^24^ Zinc chloride promoted coupling of **3** with enol ether **4** produced **5** in low yield. Removal of the TBS protecting group with HF, followed by rhodium acetate ring closure produced bicyclic ketoester **7**. Conversion of **7** to the corresponding enol phosphate, followed by coupling with thiol **8** produced the protected carbapenem **9**. Catalytic hydrogenolysis of the two *p*-nitrobenzyl protecting groups in a biphasic system of ethyl acetate and pH 6 phosphate buffer produced JDB/PQ-1-219 in low yield. JDB/PQ-1-219 was purified on Diaion HP-20SS polymeric resin. Purity was evaluated by UPLC at >95% (Figure S1).

**Scheme 1:**
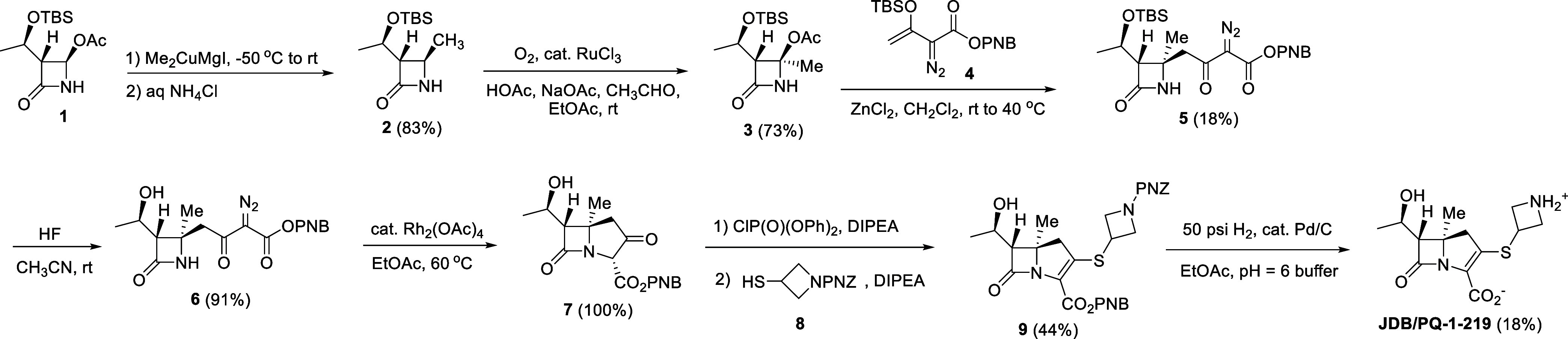
Synthesis of JDB/PQ-1-219

### Antibiotic Susceptibility and Cytotoxicity Testing

The efficacy of JDB/PQ-1-219 was tested against a collection of isogenic *A. baumannii* CIP 70.10 strains expressing various CHDLs belonging to the three molecular classes and also against MDR clinical isolates from the CDC and FDA Antibiotic Resistance (AR) Isolate Bank. The MICs were compared to the commercial carbapenem Meropenem and the atypical carbapenem NA-1-157, which differs from JDB/PQ-1-219 in its C2 substituent (Figure 1). While the MIC value of JDB/PQ-1-219 (1 μg/mL) against the parental strain was 2-fold higher than that of Meropenem (0.5 μg/mL), its MICs against the isogenic strains expressing the major *A. baumannii* carbapenemase OXA-23 or other CHDLs, OXA-24/40, −51M, −58, and −143, were 64-, 64-, 16-, 4-, and 128-fold lower, respectively (Table 1). MICs of JDB/PQ-1-219 against class A (GES-5) and class B (NDM-1) carbapenemases were 32-fold lower than those of Meropenem. Importantly, the resulting MICs of JDB/PQ-1-219 were all in the clinically susceptible range (≤2 μg/mL).^25^ Unlike for JDB/PQ-1-219, MICs of NA-1-157 were in the clinically sensitive level only for the isolates producing CHDL OXA-58 (a relatively weak carbapenemase) and class A carbapenemase GES-5, while strains producing the CHDL OXA-143 and class B carbapenemase NDM-1 were resistant to the antibiotic. A similar trend was observed for 44 clinical isolates producing various CHDLs and other β-lactamases (Table 2 and Figure S2). While MICs of JDB/PQ-1-219 against all of these strains were in the clinically sensitive level, those of NA-1-157 varied from susceptible to resistant range, precluding the clinical utility of the latter. Antibiotic sensitivity testing demonstrated that substitution of the C2 (dimethylaminocarbonyl)­pyrrolidinyl group of NA-1-157 by a smaller azetidine substitution not only elevated activity of the resulting compound (JDB/PQ-1-219) against *A. baumannii* isolates producing various CHDLs to a clinically susceptible level, but also expanded the spectrum of activity to include the major metallo β-lactamase NDM-1, which is increasingly conferring resistance to carbapenems in this pathogen.^18^

**Table 1:** MICs of Selected Carbapenems against Isogenic *A. baumannii* CIP 70.10 Strains Expressing β-lactamases^a^

	MIC (μg/mL)							
carbapenems	control	class D					class A	class B
	*Ab* CIP70.10^a^	OXA-23	OXA-24	OXA-51M^b^	OXA-58	OXA-143	GES-5	NDM-1
JDB/PQ-1–219	1	1	2	2	2	2	1	2
MEM	0.5	64^c^	128^c^	32^d^	8^c^	256^c^	32^e^	64
NA-1–157	0.5	4^c^	4^c^	4	1^c^	8^c^	2	32

a Carbapenems sensitive control strain.

b OXA-51M is the W213M mutant of OXA-51.^51^.

c Data previously published.^22^.

d Data for OXA-51M previously published.^51^.

e Data for GES-5 previously published.^26^.

**Table 2:** MICs of Selected Carbapenems Against MDR Clinical *A. baumannii* Isolates^a^

	MIC (μg/mL)			
enzyme^a^	number of strains	meropenem^b^	NA-1–157^b^	JDB/PQ-1–219
OXA-23	22	32–128	2–4	1–2
OXA-24/40	8	128–256	2–4	2
OXA-72 (OXA-24/40-like)	7	256–512	4–8	2
OXA-58	2	8	2	1–2
OXA-237 (OXA-134-like)	2	16–32	2–4	2
OXA-51-like^c^	3	16–32	2–4	2

a All strains encode one of the chromosomal OXA-51-like enzymes (such as OXA-65, −66, −69, −82, −100, −104, −223). The class A β-lactamases PER-7, SHV-5 and TEM-type are encoded in 1, 1, and 20 strains, respectively. The class C enzyme ADC-25 is encoded in 25 strains.

b Data were previously published.^23^.

c OXA-51 variants (OXA-71 with SHV-5; OXA-82 with TEM-type and ADC-25).

Given the broad spectrum clinically relevant activity of JDB/PQ-1–219, the compound was tested for cytotoxicity against HepG2 human liver cancer cells. The compound was not toxic up to the highest concentration tested (500 μg/mL; 1.6 mM), which is 250-fold above its MIC values with *A. baumannii* clinical strains (data not shown).

### Enzyme Kinetics

To gain insights into the interaction of JDB/PQ-1-219 with OXA-23, kinetic parameters for were measured (Table 3 and Figure S3). With an excess of JDB/PQ-1-219, the reaction stopped after acylation of the enzyme (*t*_1/2_ ∼ 10 min), indicating that the compound is an inhibitor of OXA-23. Under both steady-state and single turnover conditions, acylation of OXA-23 by JDB/PQ-1-219 was monophasic. This is different from what was previously observed with both NA-1-157 and Meropenem, where fast and slow phases of the reaction were clearly visible.^23^ However, we cannot exclude the existence of a second phase if it constitutes only a very small fraction (<5%) of the total reaction and thus would be below the detection level of the spectrophotometer. The acylation rate constant (*k*_2_) was evaluated under single turnover conditions and found to be 1.2 x 10^–3^ s^–1^, which is ∼6- and ∼47-fold slower than the same parameter for the structurally similar carbapenems NA-1-157 and Meropenem, respectively (Table 3). The maximum rate of inactivation (*k*_inact_) was determined to be 1.12 × 10^–3^ s^–1^, which is identical to the *k*_2_ value. These data suggest that *k*_2_ reflects formation of the essentially irreversible acyl-enzyme intermediate, however if the rate of acylation is limited by the rate of formation of a preacylation intermediate(s), the *k*_2_ value may be higher.^22,23,26^ The deacylation rate constant (*k*_3_) was measured discontinuously and found to be 360-fold slower than *k*_2_, showing that deacylation is the rate-limiting step in catalysis. This rate is ∼16-fold slower than that of NA-1-157,^23^ demonstrating that the OXA-23-JDB/PQ-1-219 acyl-enzyme intermediate is much more stable, with a residence time of 5050 ± 460 min. The *K*_i_ and *K*_I_ values, reflecting the binding affinity of OXA-23 for JDB/PQ-1-219 in the preacylation complex­(es) and the acyl-enzyme intermediate, respectively, were measured to be 1.1 ± 0.1 and 1.7 ± 0.3 μM (Table 3); these values are 1.9- and 2.6-fold lower than those for NA-1-157.

**Table 3:** Kinetic Parameters for OXA-23 Interaction with Carbapenems^a^

	antibiotic		
parameter	JDB/PQ-1–219	NA-1–157^a^	meropenem^a^
*k*_inact_ (s^–1^)^b^	(1.12 ± 0.06) × 10^–3^	(6.8 ± 0.3) × 10^–3^	NA^c^
*k*_cat_ (s^–1^)	NA	NA	(6.8 ± 0.1) × 10^–2^
*K*_i_ (μM)	1.1 ± 0.1	0.59 ± 0.02	NA
*K*_s_ (μM)	NA	NA	0.06 ± 0.01
*K*_I_ (μM)	1.7 ± 0.3	0.65 ± 0.07	NA
*K*_m_ (μM)	NA	NA	≤2
*k*_inact_/*K*_I_ (M^–1^s^–1^)	(6.6 ± 1.2) × 10^2^	(1.1 ± 0.1) × 10^4^	NA
*k*_cat_/*K*_m_ (M^–1^s^–1^)	NA	NA	≥3.4 × 10^4^
*k*_2 fast_ (s^–1^)	ND^d^	>26 ± 1	≥330
*k*_2 slow_ (s^–1^)	(1.20 ± 0.02) × 10^–3^ ^e^	(7.1 ± 0.4) × 10^–3^	(5.6 ± 0.1) × 10^–2^
*k*_3_ (s^–1^)	(3.3 ± 0.3) × 10^–6^	(5.5 ± 0.3) × 10^–5^	0.12 ± 0.01
residence time (min)	5050 ± 460	300 ± 20	0.14 ± 0.01

a These data were previously reported.^23^.

b For NA-1-157, this parameter was previously described as *k*_NA‑1–157_.^23^.

c NA, not applicable.

d ND, not determinable.

e Acylation was monophasic.

### Permeability Studies

Evaluation of the results of the antimicrobial susceptibility and enzyme kinetics studies shows that acylation of OXA-23 by JDB/PQ-1-219 is less efficient than that by NA-1-157 (as judged by the *k*_2 slow_ values, Table 3), however JDB/PQ-1-219 is a more efficient antibiotic (based on MIC values, Table 1). To investigate whether penetration of the bacterial cell membrane by the compounds might be *a* factor in this observed discrepancy, we determined their MICs against the hyperporinated and efflux deficient *A. baumannii*ATCC 17978 strains (Table 4).^27^ Unlike for NA-1-157 and Meropenem, whose MICs were reduced up to 4-fold, the MICs of JDB/PQ-1-219 remained unchanged. This shows that the permeability of this compound is less affected by porins and efflux pumps (as it is for the other two antibiotics), thus allowing more efficient access to its target PBPs. This could contribute to the superior activity of JDB/PQ-1-219 relative to NA-1-157.

**Table 4:** MICs of Carbapenems against *A. baumannii* ATCC 17978 and Its Hyperporinated And/or Efflux Deficient Variants^a^

	vector	overexpressed porin	Δefflux pump	overexpressed porin
				+Δefflux pump
carbapenem	*Ab*-ARA	*Ab*-ARA-EcPore	*Ab*Δ3-ARA	*Ab*Δ3-ARA-EcPore
JDB/PQ-1-219	1	1	1	1
NA-1-157	0.5	0.25	0.25	0.125
MEM	0.25	0.125	0.125	0.063

a MICs are given in μg/mL.

### Electrospray Ionization-Liquid Chromatography/Mass Spectrometry (ESI-LC/MS)

Our kinetic experiments demonstrated that acylation of OXA-23 by JDB/PQ-1-219 was monophasic. This is different from what was previously observed with interaction of OXA-23 with another C5-substituted carbapenem NA-1-157 ^23^ and also from the interaction of NA-1-157 and the commercial carbapenem Meropenem with other carbapenemases,^22,26,28,29^ where fast and slow phases of the reaction were clearly visible. To examine the nature of the products formed upon interaction of OXA-23 with JDB/PQ-1-219, they were analyzed by ESI-LC/MS under denaturing conditions. OXA-23 was incubated with an excess of JDB/PQ-1-219, and the products of their interaction at different time points (0, 10, and 60 min) were evaluated. Formation of a covalent OXA-23-JDB/PQ-1-219 complex was already complete at the earliest time point as judged by the proportional increase of the mass of OXA-23 by a mass equivalent to JDB/PQ-1-219 (Figure S4). This is similar to what was observed with another C5α-methyl carbapenem NA-1-157.^23^ OXA-23 (28,950 Da) formed three acyl-enzyme adducts with JDB/PQ-1-219 (29,248, 29,205, and 29,162 Da); the two smaller adducts may result from fragmentation of the carbapenem during MS, where either the carboxylate or 6α-HE group or both are lost.^30,31^ To follow the nature of the complexes, aliquots of the reaction were desalted at various time points to remove free JDB/PQ-1-219 and subsequently treated with an excess of NA-1-157. The spectra of adducts of OXA-23 with these two carbapenems had minimal overlap, which allowed us to distinguish their complexes from each other. This experiment demonstrated that the majority (∼90%) of the covalent OXA-23-JDB/PQ-1-219 complexes were reversible as judged by the presence of OXA-23-NA-1-157 complexes (Figure S4). As the residence time of the OXA-23-JDB/PQ-1-219 acyl-enzyme intermediate is ∼84 h, this reversible complex must be a covalent preacylation intermediate(s) as previously described.^22,23,26^ Over time, the amount of the OXA-23-NA-1-157 complexes decreased, reflecting an increase in formation of the kinetically stable OXA-23-JDB/PQ-1-219 acyl-enzyme intermediate. ESI-LC/MS experiments, combined with kinetic studies, revealed that unlike for interaction of OXA-23, OXA-58, and GES-5 with NA-1-157, where formation of both, acyl-enzyme (fast phase) and reversible preacylation complexes (slow phase) were detected, interaction of OXA-23 with JDB/PQ-1-219 yielded only formation of the latter, reflected by the observed monophasic nature of the kinetics.

### Time-dependent in Crystallo Acylation of OXA-23 by JDB/PQ-1-219

The kinetic differences observed for the interaction of OXA-23 with JDB/PQ-1-219 and NA-1-157, including the monophasic versus biphasic acylation behavior and the ∼6-fold difference in acylation rate, were unexpected given the close similarity of the two molecules (the only difference being the composition of the C2 tail group; Figure 1). Recent structural and time-resolved studies of NA-1-157 bound to OXA-23 ^23^ revealed that inhibitor binding perturbs access of a deacylating water and induces partial decarboxylation of the catalytic carboxylated Lys73 (hereinafter designated Lys73^CO2^, with residue numbering based on the SAND numbering scheme^32,33^ ). To investigate the structural response of OXA-23 upon JDB/PQ-1-219 binding, and to determine whether the observed kinetic differences arise from distinct enzyme–inhibitor complex conformations (relative to NA-1-157), we generated OXA-23-JDB/PQ-1-219 complexes. Because acylation of OXA-23 by JDB/PQ-1-219 proceeds substantially more slowly than that by NA-1-157, eight soaking time points (versus only three for NA-1-157 acylation) ranging from 3 to 90 min were examined (Table 5). This allowed the acylation trajectory and associated active site dynamics to be resolved in greater detail.

**Table 5:** Time-Resolved Acylation of OXA-23 by JDB/PQ-1-219^a^

time	resolution (Å)	6α-HE rotamer^a^	S118 Oγ-N4 distance (Å)	occupancy		
				JDB/PQ-1-219	lys73 (carboxylated/free)	leu158
						*A*/*B* ^b^
0^c^	1.40	-	-	-	1.0/0	1.0/0
3 min	1.90	-	-	-	0.88/-^d^	0.95/-^e^
5 min	1.65	-	-	-	0.85/-^d^	0.83/-^e^
10 min	1.65	II and III	3.10	0.72^f^	0.63/0.37	0.64/-^e^
20 min	1.59	II and III	3.35	0.8^g^	0.6/0.4	0.35/0.65
30 min	1.65	II	3.55	0.91	0.36/0.64	0.4/0.6
40 min	1.60	II	3.65	0.91	0.33/0.67	0.4/0.6
60 min	1.57	II	3.85	1.0	0/1.0	0.3/0.7
90 min	1.70	II	3.90	0.91	0/1.0	0.32/0.68

a The rotamer types are named according to convention.^23,34^ .

b Two alternate rotamers (*A* and *B*) of the leucine side chain. Only one rotamer is present in substrate-free OXA-23.

c Represented by the substrate-free OXA-23 structure (PDB code 9NSW).

d The average of the three atoms (CX, CQ1 and CQ2) of the Lys73^CO2^carboxylate. The free Lys73 side chain was not modeled at these two time points.

e The average of three atoms (Cγ, Cδ1 and Cδ2) in the leucine side chain. An alternate position of the side chain was not modeled at these two time points.

f Sulfate is present with a calculated occupancy of 0.28.

g Sulfate is present with a calculated occupancy of 0.2.

The substrate-free OXA-23 structure (PDB code 9NSW), crystallized under the same conditions as the crystals used for soaking experiments, served as the reference time point. In the substrate-free structure, the active site is occupied by a sulfate anion,^23^ while Lys73 is fully carboxylated and stabilized within an internal pocket known as the catalytic lysine pocket (CLP)^15^ by hydrogen bonds with Trp157 and Ser70. Inspection of 2*F*_o_ *-F*_c_, *F*_o_ *-F*_c_ and Polder difference maps for all eight JDB/PQ-1-219 soak times (calculated after Fourier synthesis and prior to refinement) revealed time-dependent acylation of the active site Ser70. At the earliest time points (3 and 5 min; Figure S5A,B), the sulfate anion dominated the density, and inhibitor binding could not be modeled reliably. By 10 min, however, the maps (Figure 2A,B) indicated near-complete occupancy of the acyl-enzyme complex (Table 5). The inhibitor is covalently attached to Ser70 (Figure 2C), with its core adopting a canonical conformation where the O7 carbonyl oxygen is positioned in the oxyanion hole formed by the main chain amide nitrogen atoms of Ser70 and Trp211 and the C3 carboxylate forms hydrogen bonds with Thr209 and Arg250. The pyrroline ring is present as the Δ^2^ tautomer, with the C2 atom sp^2^-hybridized and the exocyclic sulfur coplanar with the ring. The azetidine tail group is weakly defined at this time point, consistent with its conformational flexibility. The 2*F*_o_ *-F*_c_ maps (Figure 2D) suggested two 6α-hydroxyethyl (6α-HE) rotamers were present, one where the O62 atom points inward and is hydrogen bonded to Lys73^CO2^ (the type-III rotamer,^23,34^ ) and a second where both the O62 and C62 atoms project outward (the type-II rotamer). By 30 min and longer, the type-II rotamer predominates (Figure S6), while density for the azetidine tail becomes progressively better defined (Figure S6).

**Figure 2:**
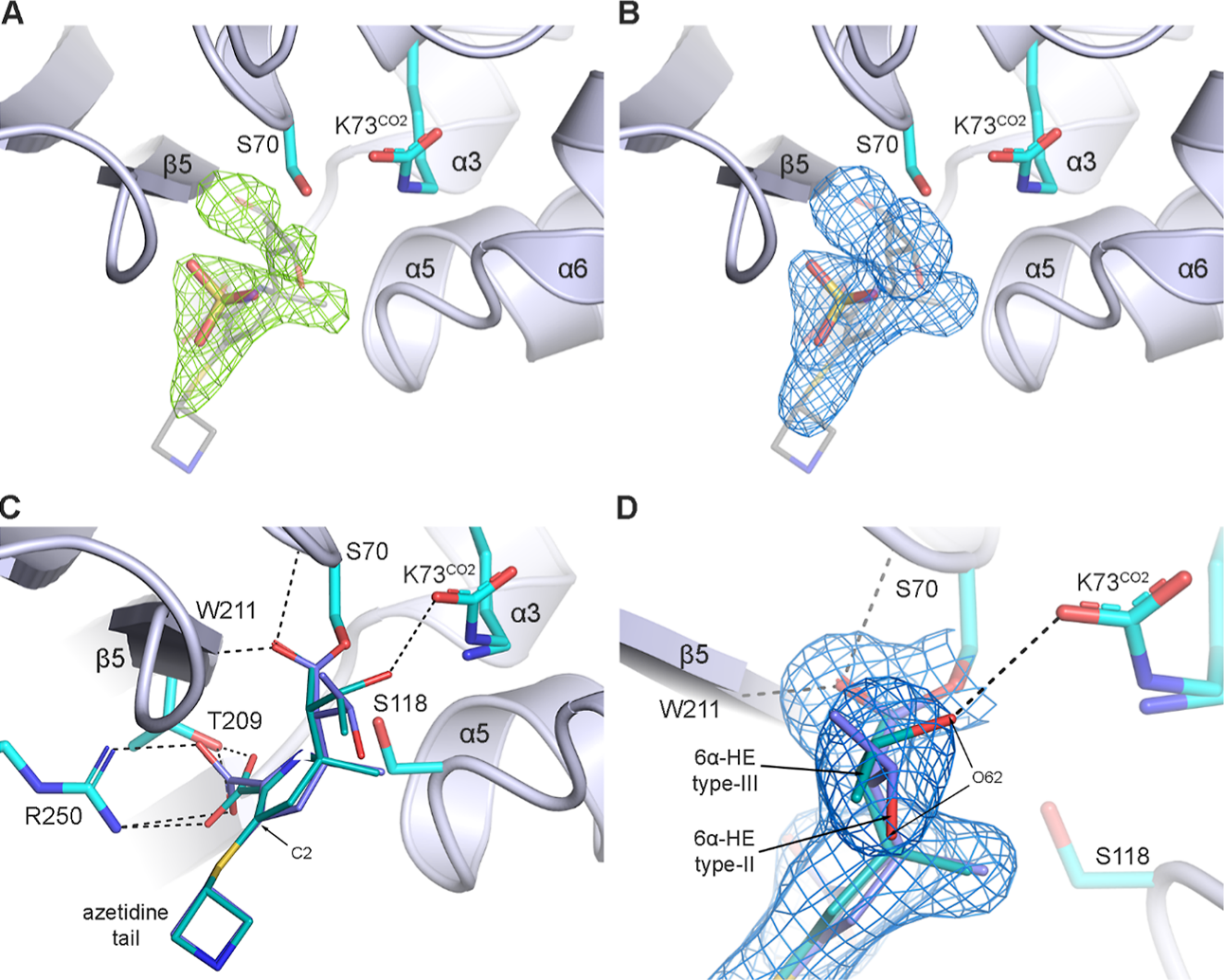
Structural analysis of the OXA-23-JDB/PQ-1-219 complex at 10 min. (A) *F*_o_ *-F*_c_ difference density map (green mesh, 3σ), clearly shows the shape of the inhibitor. (B) Polder difference map (blue mesh, 3.5σ). (C) Binding mode of JDB/PQ-1-219. Two alternate poses for the inhibitor are modeled as blue and teal sticks. (D) Final 2*F*_o_ *-F*_c_ map for JDB/PQ-1-219 showing the density for the 6α-HE group, with the two rotamers indicated.

Time-resolved analysis of the eight complexes revealed three major active site changes associated with acylation of Ser70 by JDB/PQ-1-219. The first involves progressive decarboxylation of Lys73^CO2^ ultimately leading to complete loss of the carboxylate (Table 5). At 10 min, refinement of individual atomic occupancies of the carboxylate carbon and the oxygen atom (O1) furthest from Ser70 were 0.65, while the second proximal oxygen (O2) retained full occupancy (Figure 3A). Because the carboxylate atoms must share equal occupancy, the higher occupancy at O2 indicated additional atomicity at that position (Figure 3B). The extra atomicity at O2 was modeled as water which hydrogen bonds to the free Lys73 Nζ and Trp157 Nε2 (Figure 3C). Between 10 and 60 min, the carboxylate density progressively detaches from Lys73, consistent with gradual cleavage of the Lys73-CO_2_ bond, and by 60 min only the presence of either liberated CO_2_ or water was observed. At 60 and 90 min, both a free CO_2_ molecule and a water were refined into the density with partial occupancies, likely reflecting a dynamic equilibrium between CO_2_ release and solvent replacement. This contrasts with NA-1-157 where only partial, plateauing decarboxylation at ∼60% was reported.^23^

**Figure 3:**
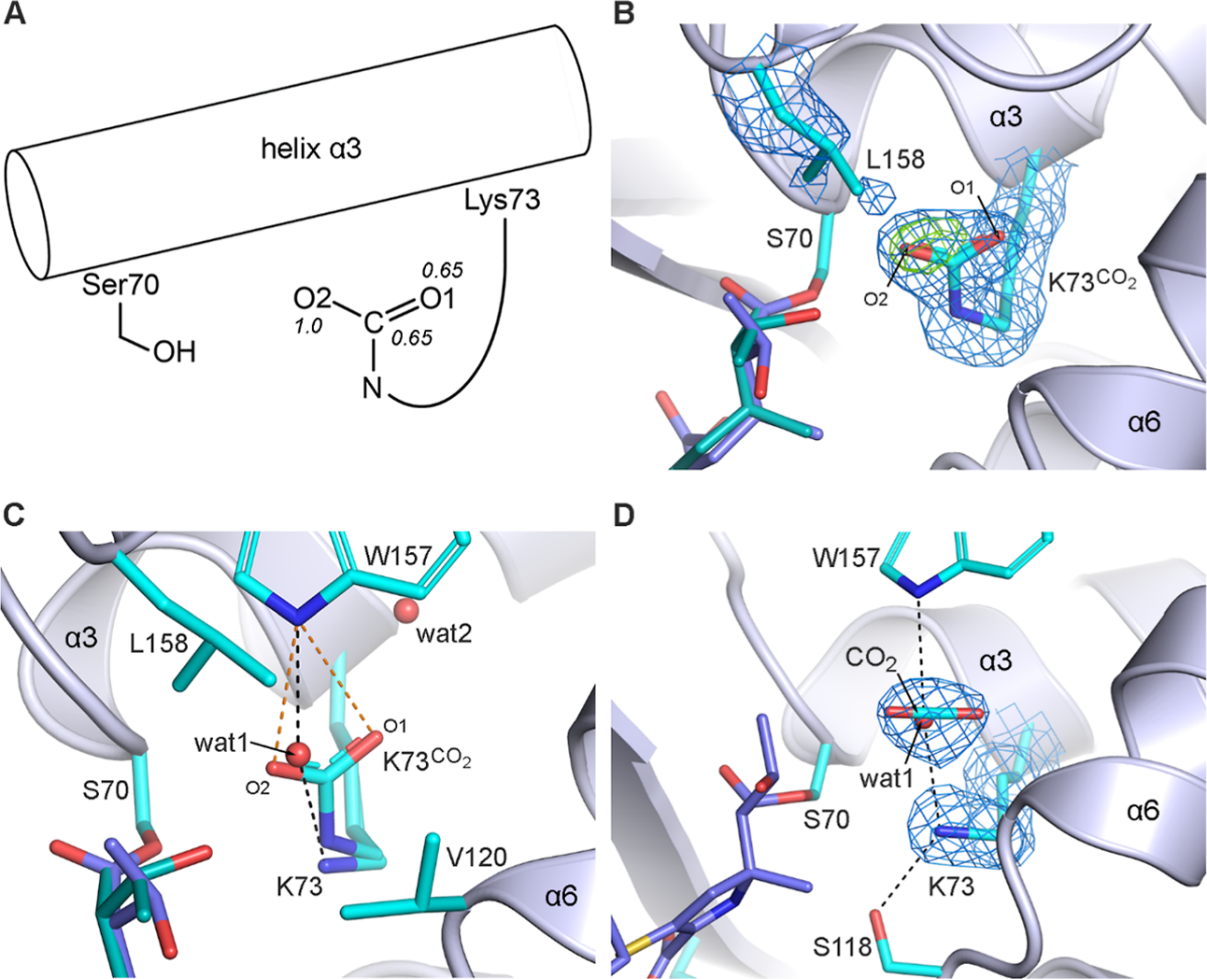
Structural analysis of the carboxylated lysine in OXA-23. (A) Schematic representation of the two catalytic residues, Ser70 and Lys73^CO2^, with the mismatch in the refined occupancy of the carboxylate group indicated. (B) 2*F*_o_ *-F*_c_ (blue mesh, 1 σ) for Lys73^CO2^ and Leu158 at 10 min. Extra atomicity at the proximal O2 atom is visible as a peak in the *F*_o_ *-F*_c_ density (green mesh, 3.5 σ) when the occupancy for all three carboxylate atoms is fixed at 0.65. (C) Partial decarboxylation at 10 min modeled as a mixture of carboxylated lysine (Lys73^CO2^), and free Lys73 plus a water molecule (wat1). (D) Full decarboxylation at 60 min modeled as free Lys73 plus water (wat1), and a liberated CO_2_ molecule. The 2*F*_o_ *-F*_c_ density for the CO_2_ and Lys73 is shown as blue mesh contoured at 1 σ.

The second structural change involves Leu158, which together with Val120 forms a hydrophobic cap that sequesters Lys73^CO2^ within the CLP. Upon JDB/PQ-1–219 binding, Leu158 progressively shifts from the preferred *mt* rotamer (using the Richardson nomenclature^35^) observed in substrate-free OXA-23 (designated Leu158A; Figure S7A) to an alternate *tp* rotamer (designated Leu158B). This transition was initially observed as weakened density at 10 min (Figure 3B, Table 5), and by 20 min (Figure S7A) both rotamers can be modeled with occupancies of 0.4:0.6, which remained stable thereafter (Table 5). Concurrently with the Leu158 conformational change, a water molecule enters into a site near the Lys73^CO2^, positioned between Leu158A and Val120 (Figure S7B). Although the same pairing of *mt* and *tp* rotamers was reported for the NA-1-157 complexes,^23^ the slower acylation of JDB/PQ-1-219 enabled the transition to be captured in greater detail.

The third structural change was revealed to be the outward movement the α5-α6 loop, based upon superposition of the eight JDB/PQ-1-219 acyl-enzyme complexes onto substrate-free OXA-23 (Figure S8). Between 10 and 90 min, the loop undergoes a progressive displacement (Figure 4A) driven by steric pressure exerted by the inhibitor C5-methyl group against Ser118, similar to that described previously for NA-1-157.^23^ This displacement propagates into the adjacent coupled P-loop and helices α5 and α6, which behave as a rigid-body structural unit (Figure 4B). As the α5-α6 loop relaxes outward, the distance between the Ser118 Oγ and the inhibitor N4 atom increases from 3.1 to 3.9 Å (Table 5), gradually disrupting the hydrogen bonding geometry required for efficient proton transfer. Since the proton withdrawn from Ser70 by Lys73^CO2^ can no longer move further through the shuttle to N4, the positive charge accumulates on the carbamate thus promoting decarboxylation. Consistent with this mechanism, progressive loss of Lys73^CO2^ carboxylate occupancy is observed over the same time period. The decreasing carboxylate occupancy is accompanied by weakening of the O62-Lys73^CO2^ hydrogen bond and accumulation of the 6α-HE type-II rotamer of bound JDB/PQ-1-219 (Figure 2D). Fundamentally, loss of the Lys73 carboxylate abolishes the general base required to activate the deacylating water molecule, thereby rendering the enzyme effectively deacylation deficient.

**Figure 4:**
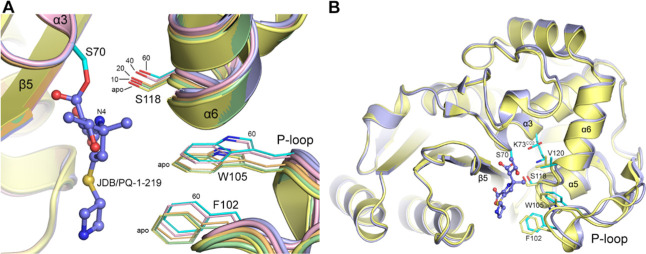
Time-resolved structures of the OXA-23-JDB/PQ-1-219 complex. (A) Superposition of OXA-23–JDB/PQ-1-219 complexes at 10 min (light green ribbons and sticks), 20 min (light orange ribbons and sticks), 40 min (pink ribbons and sticks) and 60 min (light blue ribbons and cyan sticks) onto substrate-free OXA-23 (9NSW; yellow ribbons and sticks) in the active site. The position of JDB/PQ-1-219 (blue ball-and-sticks) is shown for the 60 min time point. (B) Superposition of the 60 min OXA-23-JDB/PQ-1-219 complex (light blue ribbons and cyan sticks) onto substrate-free OXA-23 (9NSW; yellow ribbons and sticks).

It is important to emphasize that the progressive increase in the Ser118 Oγ-N4 separation and the concomitant decrease in Lys73 carboxylation do not represent an abrupt transition, but rather a continuum of partially active catalytic states. Moreover, in crystallo where the diffraction signal arises from tens to hundreds of billions of enzyme molecules, each molecule may occupy a different point along this continuum, such that each time point reflects an ensemble-averaged snapshot of the trajectory. As the reaction progresses, the electrostatic environment surrounding the catalytic serine and inhibitor becomes increasingly unfavorable for both the nucleophilic attack on the β-lactam carbonyl, and protonation of N4 during collapse of the tetrahedral intermediate. The cumulative effect is a gradual, time-dependent erosion of catalytic proficiency. Acylation can still occur as long as in some OXA-23 molecules Lys73 remains carboxylated, as evidenced by the buildup of inhibitor occupancy. However, progressive decarboxylation steadily weakens the catalytic network, driving the enzyme toward increasingly decoupled and less catalytically competent states.

Given the strict conservation of sequence, structural architecture, and catalytic mechanism across CHDLs characterized to date,^15,36–38^ the JDB/PQ-1-219 induced decarboxylation of Lys73 observed in these time-resolved structures appears directly associated with enzyme inhibition, and may represent a general CHDL inactivation mechanism for this modified carbapenem class. Alternative inhibitory mechanisms proposed for β-lactamases, including steric occlusion of the active site, stabilization of long-lived acyl-enzyme intermediates, or active-site rearrangements alone, do not readily account for the sustained loss of catalytic activity of OXA-23 and prolonged residence times observed for JDB/PQ-1-219. Together, the time-resolved structural data support a mechanism in which Lys73 decarboxylation and associated α5-α6 loop reorganization drive functional inactivation of OXA-23 by JDB/PQ-1-219.

## Conclusions

In this study we describe the synthesis and characterization of the novel carbapenem JDB/PQ-1-219. The antibiotic has broad spectrum activity against *A. baumannii*isolates producing carbapenemases from three molecular classes (A, B and D). The MICs of JDB/PQ-1-219 against various strains of the pathogen which are highly resistant to all commercial carbapenems, are all within the clinically susceptible range (≤2 μg/mL). Unlike with commercial carbapenems and the C5 methyl-substituted carbapenem NA-1-157, penetration of JDB/PQ-1-219 through bacterial outer membrane is less impeded by bacterial porins and efflux pumps. The compound showed no toxicity at the highest concentration tested (250-fold its MIC against *A. baumannii* clinical strains). Detailed studies with the most clinically important *A. baumannii* carbapenemase, OXA-23, showed that in contrast to other existing carbapenems, formation of the acyl-enzyme OXA-23-JDB/PQ-1-219 intermediate is monophasic and proceeds via a reversible tetrahedral intermediate. Multiple structural snapshots along the acylation trajectory allowed for molecular level observation of dynamics changes associated with acylation, ultimately leading to decarboxylation of the catalytic lysine residue and long-lived inhibition of the enzyme. Combined, these results demonstrated that JDB/PQ-1-219 is a potential candidate for subsequent preclinical studies as a promising novel carbapenem antibiotic for the treatment of infections caused by multidrug and pan-drug resistant *A. baumannii*.

## Materials and Methods

### General Methods

All starting materials, reagents, and solvents were obtained from commercial vendors and used as received without further purification. NMR spectra (^1^H and ^13^C) were recorded on a 400 MHz Bruker AVANCE DRX Multinuclear NMR spectrometer and chemical shifts are reported in ppm relative to TMS. IR spectra were measured on a PerkinElmer model 1600 FTIR. HRMS were measured on a Shimadzu LCMS-9030 Q-TOF Mass Spectrometer. Compound purity was assessed by ultraperformance liquid chromatography–mass spectrometry, using a (UPLC–MS) using a Waters ACQUITY UPLC H-Class system coupled to a Bruker Impact II ultrahigh-resolution Qq-TOF mass spectrometer controlled with Hystar 5.0 SR1 software. JDB/PQ-1-219 was shown to be >95% pure (Figure S8).

### Antibiotic Susceptibility and Cytotoxicity Testing

MIC determination was performed in 96 well plates in cation adjusted Muller Hinton II broth according to the CLSI guidelines.^25^ For *A. baumannii* ATCC 17978 and its hyperporinated and efflux-deficient derivatives (*Ab*-ARA; *Ab*-ARA-EcPore, *Ab*Δ3-ARA, *Ab*Δ3-ARA-EcPore,^27^ the growth medium was supplemented with 0.1% L-arabinose.^39^

The toxic potential of JDB/PQ-1-219 was tested with the XTT Cell Proliferation Assay Kit (Canvax) using HepG2 human liver cancer cells. On a 96 well plate, the cells were treated with various concentrations of JDB/PQ-1–219 (2–500 μg/mL) for 24 h of incubation, and the cellular metabolic activity was colorimetrically measured according to the manufacturer’s procedure.

### Purification of OXA-23

The OXA-23 β-lactamase was expressed and purified as reported earlier.^23,40^ Protein concentration was determined by measuring the OD_280_ (MW = 28,950 Da, ε = +40,500 M^–1^cm^–1^).

### Enzyme Kinetic Studies

Data were collected using a Cary 60 spectrophotometer (Agilent) at 22 °C. Reactions were measured in 100 mM sodium phosphate, pH 7.0 containing 50 mM NaHCO_3_ unless otherwise stated. Data analysis was completed using Prism 10 (GraphPad Software, Inc.).

### Steady-State Reactions

Reactions containing various concentrations (10–100 μM) of JDB/PQ-1–219 (Δε 308 nm = −8100 M^–1^cm^–1^) were initiated by the addition of OXA-23 (0.1–10 μM).

### Determination of the Dissociation Constant K_i_ and Inactivation Parameters *K*_inact_ and K_I_

In the competition experiment nitrocefin (Δε 500 nm = 15,900 M^–1^cm^–1^) was used as a reporter substrate. The reaction buffer contained 320 μM nitrocefin, 0.2 mg/mL BSA, varying concentration of JDB/PQ-1-219 (0–27 μM), and started by addition of 0.052 nM OXA-23. The progress curves were recorded continuously and analyzed with the time-dependent equation to determine the initial velocities and *k*_inter_ values. The initial velocities were graphed as a function of JDB/PQ-1-219 concentration and fitted to the Morrison equation to calculate the dissociation constant *K*_i_. The *k*_inter_ values were plotted against the concentrations of the JDB/PQ-1-219, and the data were fitted to the equation, which includes the correction to take account of the competitive nature of the reporter substrate, to calculate the inactivation rate constant *k*_inact_.

$$k_{inter}=\frac{k_{inact}\left[I\right]}{K_{I}\left(1+\frac{S}{K_{m}}\right)+\left[I\right]}$$

In this equation S is the nitrocefin concentration, *K*_m_ is the Michaelis constant of nitrocefin, and I is the concentration of JDB/PQ-1-219, *k*_inact_ is the maximum rate of inactivation, and *K*_I_ represents the inhibitor concentration that is needed to reach one-half of the inactivation rate.

### Determination of the Acylation Rate Constant, *k*_2_

Changes in absorbance over time were measured at 308 nm under single turnover conditions, where OXA-23 (25 and 50 μM) was in 5 to 10-fold molar excess over JDB/PQ-1-219, correspondingly. Progress curves of the reactions were fitted to the one phase exponential decay equation to calculate the acylation rate constant, *k*_2_.

$$A_{t}=\left(A_{0}-A_{\infty }\right)e^{\left(-k_{2}t\right)}+A_{\infty }$$

where *A*_0_ is the initial absorbance, *A*_*t*_ is the absorbance at time *t* of the reaction, *A*_∞_ is the absorbance at infinite time, and *k*_2_ is the acylation rate constant.

### Determination of the Deacylation Rate Constant, *k*_3_

The reaction buffer containing 0.2 mg/mL BSA and 2 μM OXA-23 with or without 20 μM JDB/PQ-1-219 was incubated to complete acylation. Excess compound was removed by 7 kDa cut off desalting Zeba column (Thermo Scientific). These samples were rapidly diluted 100-fold into the reaction buffer containing 0.2 mg/mL BSA from where aliquots were taken at different time points (0–400 h) to measure enzyme activity with nitrocefin (200 μM final concentration). The percentage of recovered enzyme activity (relative to 100% activity of the control reaction without inhibitor) was plotted against time. Data were fit to the exponential equation and the deacylation rate constant was calculated as previously described.^26^

$$Y_{t}=Y_{\infty }\left({1-e}^{\left(-k_{3}t\right)}\right)$$

where *Y*_*t*_ is the percentage of recovered activity at time *t* in discontinuous assay, Y_∞_ is the final percentage of activity, and *k*_3_ is the deacylation rate constant.

### ESI-LC/MS

From the reactions with or without JDB/PQ-1-219 (20 μM) and OXA-23 (2 μM) in 100 mM sodium phosphate pH 7.0, 50 mM NaHCO_3_ aliquots were taken at 0, 1, 15, 30 min, and 18 h time points and analyzed by mass spectrometry. For competition experiments, from the 400 μL reactions with or without JDB/PQ-1-219 and OXA-23 at 0, 10, 60 min and 18 h time points, 80 μL aliquots were taken and the excess of compound was removed with Zeba columns (7 kDa MWCO) according to the manufacturer’s protocol. These samples were either left untreated or further processed by taking 40 μL and adding 100 μM NA-1-157. All samples were then frozen and stored at −80 °C until analysis. As a control for the completeness of the “chase” reaction, an aliquot from the OXA-23-only reaction was passed through a Zeba column, supplemented with 100 μM NA-1-157, and stored under the same conditions.

ESI-LC/MS analysis was carried out using a DIONEX Ultimate 3000 system connected to a Bruker micrOTOF-QII mass spectrometer in positive-ion mode, utilizing Hystar 4.2 SR2 software, and data were evaluated as previously described.

### Crystallography

Crystals of OXA-23 were grown as previously described^23^ from 0.2 M Li_2_SO_4_, 0.1 M HEPES pH 7.5, 25% PEG3350. The crystals belonged to space group *P*4_2_2_1_2 with cell dimensions 83.3 × 83.3 × 85.9 Å and typically diffracted to 1.4–1.6 Å resolution. In order to remove the sulfate and facilitate the binding of JDB/PQ-1-219, a crystal stabilizing buffer comprising 0.1 M imidazole pH 7.6, 34% MPD and 25% PEG3350 was determined using established protocols.^23,41,42^ Time-resolved JDB/PQ-1-219 binding experiments were conducted by soaking substrate-free OXA-23 crystals in a 10 mM JDB/PQ-1-219 solution in stabilizing buffer for eight time points; 3, 5, 10, 20, 30, 40, 60, and 90 min. Diffraction data were collected at SSRL beamlines BL9-2 and BL12-2, and processed and scaled with XDS^43^ and AIMLESS.^44^ The structure at 3 min was solved by molecular replacement using the substrate-free OXA-23 structure crystallized under the same conditions (PDB code 9NSW). All subsequent complex structures were solved by molecular substitution (Fourier synthesis) where the partially refined 3 min OXA-23-JDB/PQ-1-219 structure (excluding JDB/PQ-1-219, sulfate and water) was used as a starting point for refinement against the associated data set using REFMAC,^45^ to ensure that all structures had the same origin. Potential model bias was removed by cycles of simulated annealing using phenix.refine.^46^ Polder omit maps were calculated for all time points using phenix.polder^47^ prior to full refinement with phenix.refine. Data collection and refinement statistics for the eight OXA-23-JDB/PQ-1-219 complexes are given in Table S1.

Superpositions of the eight JDB/PQ-1-219 complexes against substrate-free OXA-23 were initially carried out using the secondary structure matching (SSM) algorithm^48^ as implemented in COOT.^49^ Final superposition was performed with LSQKAB from the CCP4 suite^50^ using two distinct structural regions, residues 68–75 covering the conserved class D sequence *motif-1* ^15^ (PASTFK) and the two catalytic residues (Ser70 and Lys73) at the N-terminus of helix α3, and residues 207–214 from strand β5 which includes the universally conserved serine β-lactamase KTG *motif-6*.^15^ These secondary structure elements remain invariant relative to each other upon substrate and inhibitor binding and form a rigid basis for superposition thus allowing for the visualization of mobile structural elements. Structural figures were generated with PYMOL (Schrodinger) unless otherwise stated.

## Supplementary Material



## Data Availability

The structure factors and atomic coordinates for the eight OXA-23-JDB/PQ-1-219 complexes have been deposited and released in the Protein Data Bank (www.rcsb.org) with PDB codes 9ZOP, 9ZOQ, 9ZOR, 9ZOS, 9ZOT, 9ZOU, 9ZOV and 9ZOW.

## Notes

Supplemental Methods, chemical synthesis of JDB/PQ-1-219; UPLC trace of JDB/PQ-1-219 (Figure S1); MICs of selected carbapenems against clinical *A. baumannii* isolates (Figure S2); Kinetics of OXA-23 with JDB/PQ-1-219 (Figure S3); Evaluation of OXA-23-JDB/PQ-1-219 complexes by mass spectrometry (Figure S4); Early time points in the formation of the OXA-23-JDB/PQ-1-219 complex (Figure S5); The OXA-23-JDB/PQ-1-219 acyl-enzyme complex (Figure S6); The Leu158 side chain (Figure S7); Structure alignment (Figure S8); Data collection and refinement statistics for the OXA-23-JDB/PQ-1-219 complexes (Table S1); Supplemental references (PDF)

## Abbreviations

MDR: multidrug resistant
CHDL: carbapenem-hydrolyzing class D β-lactamase
PBPs: penicillin-binding proteins
6α-HE: 6α-hydroxyethyl
MIC: minimal inhibitory concentration
ESI-LC/MS: electrospray ionization-liquid chromatography/mass spectrometry
PDB: protein data bank
*rmsd*: root-mean-square deviation
CLP: catalytic lysine pocket
CLSI: clinical and laboratory standards institute
UPLC-MS: ultraperformance liquid chromatography–mass spectrometry
IR: infrared spectroscopy
HRMS: high-resolution mass spectrometry.
