# Supplementary material for: The Novel Carbapenem, JDB/PQ-1-219, Has Potent Broad Spectrum Activity against Multi-Drug Resistant Acinetobacter baumannii
Source: ACS Infect Dis. 2026 Jun 18;12(7):2396–406. doi: 10.1021/acsinfecdis.6c00415 (PMC13366572; doi:10.1021/acsinfecdis.6c00415)
Supplement: Supplementary file 1 [file id6c00415_si_001.pdf]

Supporting Information for

The Novel Carbapenem, JDB/PQ-1-219, has Potent Broad Spectrum Activity against Multi-Drug Resistant *Acinetobacter Baumannii*.

Marta Toth[a], Nichole K. Stewart[a], Ailiena O. Maggiolo[b], Pojun Quan[c], Md Mahbub Kabir Khan[c], Jonathan Cox[c], Maidileyvis Castro Cabello[c], John D. Buynak[c,*], Clyde A. Smith[b,d,*], and Sergei B. Vakulenko[a,*]

[a] Department of Chemistry and Biochemistry, University of Notre Dame, Notre Dame, IN 46556, USA

[b] Stanford Synchrotron Radiation Lightsource, Stanford University, Menlo Park, CA 94025, USA

[c] Department of Chemistry, Southern Methodist University, Dallas, TX 75275, USA

[d] Department of Chemistry, Stanford University, Stanford, CA 94305, USA

[*] Corresponding authors:

Prof. John D. Buynak, ph: 214-768-2484, Fax: 214-768-4089, E-mail: jbuynak@smu.edu

Dr. Clyde A. Smith, ph: 650-926-8544, Fax: 650-926-3292, E-mail: csmith@slac.stanford.edu

Prof. Sergei B. Vakulenko, ph: 574-631-2935, Fax: 574-631-6652, E-mail: svakulen@nd.edu

SUPPLEMENTAL METHODS

Chemical synthesis of JDB/PQ-1-219

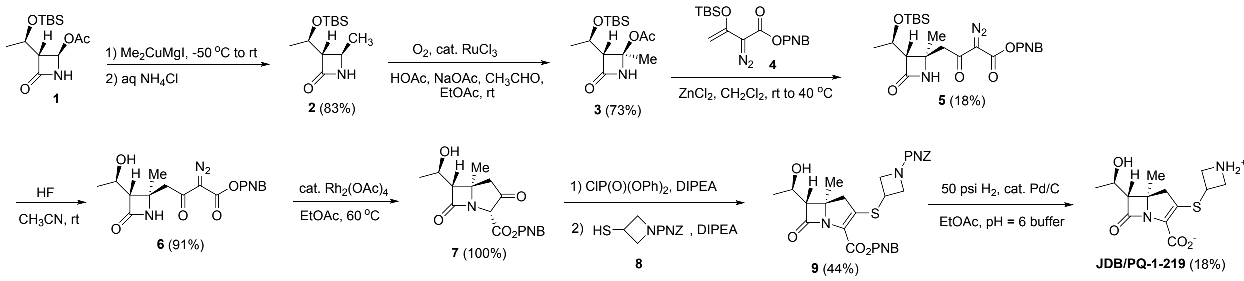

(3*S*,4*R*)-3-((*R*)-1-((tert-butyldimethylsilyl)oxy)ethyl)-4-methylazetidin-2-one (2): A solution of methyl magnesium iodide was prepared as follows. To a stirred slurry of magnesium turnings (30.5 g, 1.26 mol, 1.5 eq) in anhydrous diethyl ether was added a small crystal of iodine, and then a solution of methyl iodide (118.6 g, 52 mL, 0.835 mol) in 50 mL of anhydrous diethyl ether dropwise at such a rate as to maintain a gentle reflux. Once complete, the reaction was allowed to stir under an inert atmosphere at room temperature overnight.

To a rapidly stirred (overhead stirrer) slurry of copper (I) iodide (79 g, 0.42 mol) in anhydrous THF (2 L) at room temperature was added dimethyl sulfide (25.8 g, 30.7 mL, 0.42 mol). This solution was allowed to stir under an inert atmosphere for 30 min, then chilled to -60 °C. To this rapidly stirred solution was added the ethereal solution of methyl magnesium iodide prepared above, at a rate to maintain the temperature below -40 °C. Then the temperature of the reaction mixture was allowed to rise to between -10 and 0 °C, and stirred at that temperature for 30 min. The solution was then again cooled to -60 °C and a solution of **1** (60 g, 0.208 mol) in 300 mL anhydrous THF was added slowly. The reaction was then allowed to warm to room temperature over the course of 90 min. The reaction was again chilled to 0 °C and the remaining organometallic quenched by slowly pouring the reaction into a rapidly stirred saturated aqueous solution of ammonium chloride. The THF was then removed *in vacuo*, water was added, and the product

extracted with ethyl acetate. The combined ethyl acetate layers were washed with dilute aqueous ammonium hydroxide to remove copper. The organic layers were dried over Na_2SO_4, concentrated in vacuo, and the product purified by silica gel chromatography (increasing 2 to 40% ethyl acetate /CH_2Cl_2) to produce **2** (42.2 g, 83% yield) as a white solid. ^{1}H NMR (400 MHz, $CDCl_3$): δ 6.38 (s, 1H), 5.29 (s, 1H), 4.19 (m, 1H), 3.83 (m, 1H), 2.69 (m, 1H), 1.3 (dd, J= 54 Hz, 3H), 0.86 (s, 9H), 0.067 (S, 6H). ^{13}C NMR (100 MHz, $CDCl_3$): δ 168.99, 65.74, 65.50, 47.88, 25.74, 25.59, 22.33, 20.59, 17.77, -4.34, -4.62, -4.75; IR: 3415.79, 3229.86, 2959.39, 2929.27, 2893.85, 2857.09, 2708.17, 2249.20, 1754.52, 1471.48, 1462.92, 1446.72, 1378.34, 1347.43, 1333.39, 1299.97, 1254.06, 1187.16, 1143.14, 1096.71, 1037.92, 1005.93, 986.68, 956.49, 909.25, 835.17, 809.60, 766.45, 734.96, 661.74, 646.36 cm^{-1}; HRMS: calculated $C_{12}H_{26}NO_2Si^+$ $[M + H]^+$ 244.1727, observed 244.1686.

(2*R*,3*R*)-3-((*R*)-1-((tert-butyldimethylsilyl)oxy)ethyl)-2-methyl-4-oxoazetidin-2-yl acetate (3): To a solution of **2** (5.0 g, 20.5 mmol) in anhydrous ethyl acetate (205 mL) was added anhydrous sodium acetate (1.01 g, 12.3 mmol, 0.6 eq) and anhydrous acetic acid (10.25 mL, 10.8 g, 179 mmol, 8.7 eq). Commercial $RuCl_3$ was placed in a flask with a Teflon coated stir bar, put under high vacuum (0.1 mm Hg), and the flask heated with a Bunsen burner with external manual agitation until last traces of water were removed and the consistency was that of finely divided free-flowing black powder (approximately 15 min). This dried $RuCl_3$ (0.3 g, 1.44 mmol, 0.07 eq) was allowed to cool to rt and then added to the reaction vessel. The flask was then sealed tightly with a wired septum and the flask placed under dynamic oxygen pressure (12 psi) using a pressurized needle through the septum and chilled in an external bath to 10-12 ^{o}C. Freshly (twice) distilled acetaldehyde (11.8 g, 15 mL, 268 mmol, 13.4 eq) was then added using a chilled syringe. The reaction was then allowed to stir at 12 ^{o}C while maintaining external pressure of oxygen and

monitored by ^{1}H NMR. The reaction completed in 1-2 h and was then diluted with cold hexane and the hexane layer washed with ice cold brine until the pH of the aqueous layer reached 7 (approximately 7 to 10 washes). The organic layer was dried over Na_2SO_4 and evaporated *in vacuo* to afford **3** (4.5 g, 73% crude yield) as a purple oil. This material was unstable toward further purification and was directly used in the next reaction. ^{1}H NMR (400 MHz, $CDCl_3$): δ 7.05 (S, 1H), 4.31 (m, 1H), 3.05 (d, J= 9.2 Hz, 1H), 2.04 (s, 3H), 1.82 (s, 3H), 1.33 (d, 6Hz, 3H), 0.86 (s, 9H), 0.067 (s, 6H). ^{13}C NMR (400 MHz, $CDCl_3$): δ 170.22, 166.39, 88.61, 70.25, 68.75, 67.32, 64.69, 25.49, 21.90, 19.69, 17.64, 0.82, -3.98, -4.41; IR: 3327.71, 2957.92, 2931.39, 2887.40, 2858.22, 2253.32, 1781.31, 1472.47, 1463.20, 1416.50, 1362.49, 1254.34, 1222.80, 1171.03, 1092.37, 1015.43, 963.78, 914.20, 835.01, 812.13, 778.15, 733.78, 647.79 cm^{-1}.

4-Nitrobenzyl 4-((2*R*,3*S*)-3-((*R*)-1-((tert-butyldimethylsilyl)oxy)ethyl)-2-methyl-4-oxoazetidin-2-yl)-2-diazo-3-oxobutanoate (5): To a solution of **3** (3.0 g, 9.95 mmol) and TBS enol ether **4**[1] (5.9 g, 15.6 mmol, 1.57 eq) in 25 mL dry CH_2Cl_2 was added a solution of $ZnCl_2$ in diethyl ether (7.3 mL, 1 M, 7.3 mmol, 0.7 eq) and the flask was heated to reflux. The reaction was monitored by ^{1}HNMR, and once completed (30 min), the reaction was cooled to room temperature and diluted with ethyl acetate. The solution was washed with saturated aqueous $NaHCO_3$ once and the aqueous layer extracted with ethyl acetate twice. The combined organic layers were dried over Na_2SO_4 and then evaporated *in vacuo*. The crude material was purified by silica gel flash chromatography via gradient elution (2.5:97.5 ethyl acetate/CH_2Cl_2 to 40/60 ethyl acetate/CH_2Cl_2) to afford **5** (0.92 g, 18% yield) as a white solid. ^{1}H NMR (400 MHz, $CDCl_3$): δ 7.92 (dd, J= 287 Hz, 8.4 Hz 4H), 6.38 (s, 1H), 5.35 (d, J= 14Hz, 2H), 4.27 (t, J= 2.8 Hz, 1H), 3.59 (dd, J= 292 Hz, 16.8 Hz, 2H), 2.85 (s, 1H), 1.538 (s, 3H), 1.40 (d, J=12, 3H), 1.33 (t, J= 13.6 Hz, 2 H), 0.085 (s, 9H), 0.866 (S, 6H); ^{13}C NMR (100 MHz, $CDCl_3$): 189.84, 167.09, 160.56, 147.85, 141.95, 128.66,

128.56, 123.88, 123.81, 66.80, 65.43, 65.21, 55.71, 49.90, 25.69, 25.33, 22.29, 21.15, 19.78, 17.76, -3.30, -4.82; HRMS: calculated $C_{23}H_{33}N_4O_7Si^+$ $[M + H]^+$ 505.2113, observed 505.2836.

4-Nitrobenzyl 2-diazo-4-((2*R*,3*S*)-3-((*R*)-1-hydroxyethyl)-2-methyl-4-oxoazetidin-2-yl)-3-oxobutanoate (6): To a solution of **5** (2g, 3.96 mmol) in 20 mL of acetonitrile, 2 mL of HF (48% aqueous) was added. The reaction was stirred at room temperature and monitored by thin layer chromatography and ^{1}HNMR. If needed, additional HF was added to ensure completion in 1-3 h. Once complete, the reaction was further diluted with 100 mL ethyl acetate, and finely ground $NaHCO_3$ was carefully added to the reaction (*caution* CO_2 evolution) to attain pH = 7. The reaction was filtered to remove the precipitated NaF and evaporated *in vacuo* to afford **6** (1.4 g, 91% yield) as white solid. ^{1}H NMR (400 MHz, $CDCl_3$): δ 7.91 (dd, J= 280 Hz, 8.3 Hz, 4H), 5.34 (q, J=18.4 Hz, 13.2 Hz, 2H), 4.84 (s, 1H), 4.39 (m, 1H), 3.22 (d, J= 10` Hz, 1H), 2.7 (dd, J= 44.4, 18 Hz, 2H), 1.63 (s, 3H), 1.42 (d, J= 6.4 Hz, 3H), 0.89 (s, 1H), 0.085 (s, 2H); ^{13}C NMR (100 MHz, $CDCl_3$): δ 190.53, 167.18, 160.14, 147.43, 141.86, 128.37, 123.50, 65.32, 63.51, 54.89, 49.67, 49.07, 48.64, 48.00 21.24, 20.29; IR: 3362.70, 2969.02, 2143.11, 1722.43, 1648.53, 1522.75, 1347.90, 1306.60, 1216.14, 1127.81, 1025.25, 853.56, 739.51 cm^{-1}.

4-Nitrobenzyl (2*R*,5*R*,6*S*)-6-((*R*)-1-hydroxyethyl)-5-methyl-3,7-dioxo-1-azabicyclo-[3.2.0]heptane-2-carboxylate (7): To a solution of **6** (1.3g, 3.33 mmol) in 50 mL dry ethyl acetate was added a catalytic amount of $Rh_2(OAc)_4$ (15 mg, 0.034 mmol, 0.01 eq). The reaction was warmed to 60 °C for 30 min while monitoring by ^{1}HNMR. Once completed, the reaction was cooled to room temperature and the solvent was evaporated *in vacuo* to produce 1.21 g (100% crude yield) of **7**. This material was unstable toward further purification and was used directly in the next step. ^{1}H NMR (400 MHz, $CDCl_3$): δ 7.96 (dd, J= 232 Hz, 8.4 Hz, 4H), 5.31 (q, J= 19.6

2H), 4.15 (q, J= 8 Hz, 1H), 3.68 (q, J= 10 Hz, 1H), 3.18 (d, J= 4Hz, 1H), 2.65 (dd, J= 40 Hz, 20 Hz, 1H)), 2.08 (s, 3H), 1.55 (dd, J= 30 Hz, 15 Hz, 3H), 1.45 (d, J= 15 Hz, 1H), 1.28 (m, 1H).

4-Nitrobenzyl (5*R*,6*S*)-6-((*R*)-1-hydroxyethyl)-5-methyl-3-((1-(((4-nitrobenzyl)oxy)-carbonyl)azetidin-3-yl)thio)-7-oxo-1-azabicyclo[3.2.0]hept-2-ene-2-carboxylate (9): A solution of **7** (0.8 g, 0.0022 mol) in 10 mL of dry CH_3CN under inert atmosphere was cooled to -35 °C. Diphenyl phosphoryl chloride (0.618 g, 0.475 mL, 0.0022 mol, 1 eq) was then added to the flask, followed by a slow addition of N,N-diisopropylethylamine (0.297 g, 0.4 mL, 0.0022 mol, 1 eq), and the reaction was allowed to stir for 30 minutes, monitoring by TLC, to generate the intermediate enol phosphate, which was not isolated. Once the enol phosphate had formed, thiol **8**[2] (0.62 g, 0.0022 mol, 1 eq) and an additional 1 eq of DIPEA (0.297 g, 0.4 mL, 0.0022 mol, 1 eq) were added. The reaction was then allowed to warm to room temperature over a course of 1 h, while monitoring by ^{1}H NMR and TLC. Once completed, the reaction was diluted with ethyl acetate (200 mL) and successively washed with saturated aqueous $NaHCO_3$ and saturated aqueous NH_4Cl. The resultant ethyl acetate solution was then dried over Na_2SO_4, evaporated *in vacuo*, and further purified by column chromatography using an increasing gradient of $MeOH/CH_2Cl_2$ (0% to 10% in 1% increments) as eluent to afford **9** (0.7 g, 87.5% yield) as a white solid. ^{1}H NMR (400 MHz, $CDCl_3$): δ8.25 (d,4H), 7.8(dd, 4H), 7.2(m, 1H), 5.45(d, 1H), 5.25(d, 3H), 4.5(s, 2H), 4.2(s,1H), 4.0(s, 3H), 3.2(t,2H), 2.85(d, 1H), 2.0(s,1H), 1.7(s,3H), 1.5(s, 3H); ^{13}C NMR (100 MHz, $CDCl_3$): δ 174.89, 160.76, 155.12, 147.54, 147.47, 145.80, 143.43, 142.90, 129.67, 128.14, 128.00, 125.11, 123.69, 123.65, 123.13, 120.22, 120.17, 77.32, 77.00, 76.68, 67.93, 65.42, 65.19, 64.71, 61.22, 56.56, 48.75, 32.51, 22.51, 21.30, 0.89; IR 3435, 3113, 3080, 2967, 2883, 1771, 1704, 1607, 1494 cm^{-1}.

(5*R*,6*S*)-3-(azetidin-1-ium-3-ylthio)-6-((*R*)-1-hydroxyethyl)-5-methyl-7-oxo-1-azabicyclo[3.2.0]hept-2-ene-2-carboxylate (JDB/PQ-1-219): A two-phase solution of **9** (700 mg, 1.14 mmol) in 40 mL of ethyl acetate and 40 mL of pH 6 aqueous sodium phosphate buffer solution (0.1 M) was placed in a 250 mL Parr hydrogenation vessel. This solution was degassed by bubbling argon through it for 5 min, then 10% Pd on carbon (0.7 g) was added. The vessel was connected to the Parr apparatus, further degassed by two successive evacuation-argon repressurization cycles, then evacuated once more and subjected to hydrogen pressure at 50 psi and shaken for 90 min at this pressure. The hydrogen gas was completely removed under vacuum, the vessel opened to air, the resultant solution was filtered through celite to remove the catalyst. The filtrate was placed in a separatory funnel and the aqueous (product) layer was separated. The aqueous layer was further washed with diethyl ether to remove traces of ethyl acetate and remaining unionized organic material. Then the aqueous layer was further subjected to vacuum (rotary evaporator) to remove last traces of diethyl ether and to partially reduce the total volume. The aqueous layer was then placed on a column of Diaion CHP20P resin and eluted with increasing percentage of ethanol/water (0 to 40% in 5% increments). Tubes containing the product were identified by inspection of the UV of each fraction, those tubes displaying the carbapenem 296 nm absorption were combined, and the solvent was removed *in vacuo*. The remaining aqueous solution was transferred to a small vial, frozen, and lyophilized overnight to produce the purified carbapenem antibiotic JDB/PQ-1-219 (62 mg, 18% yield) as a white solid. ^{1}H NMR (400 MHz, $CDCl_3$): δ 4.6 (s, 2H), 4.4 (t, 1H), 4.3(t, 1H), 4.1 (q, 2H), 3.3 (q, 2H), 2.7 (d, 1H), 1.5 (s, 3H), 1.3 (d, 3H); IR 3372, 2969, 1747, 1582, 1380, 1242 cm^{-1}; HRMS: calculated $C_{13}H_{19}N_2O_4S^+$ [M + H]$^+$ 299.1060, observed 299.1068.

SUPPORTING FIGURES AND TABLES

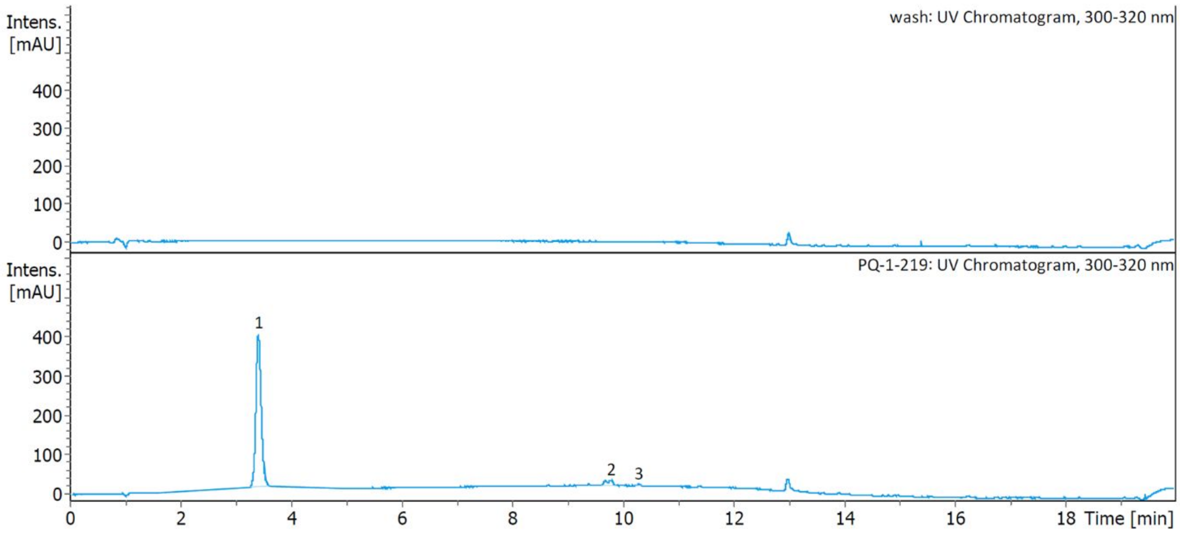

#	RT [min]	Area	Trace	Area Frac. %
1	3.4	2592.193	UV Chromatogram, 300-320 nm	95.58
2	9.8	104.901	UV Chromatogram, 300-320 nm	3.87
3	10.3	15.050	UV Chromatogram, 300-320 nm	0.55

Figure S1. UPLC trace of JDB/PQ-1-219. The main peak eluting at 3.4 min is JDB/PQ-1-219.

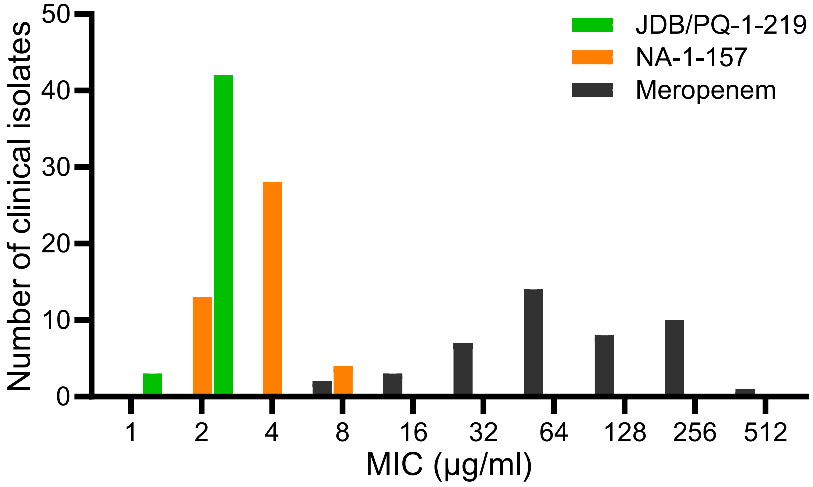

Figure S2. MICs of selected carbapenems against clinical *A. baumannii* isolates.

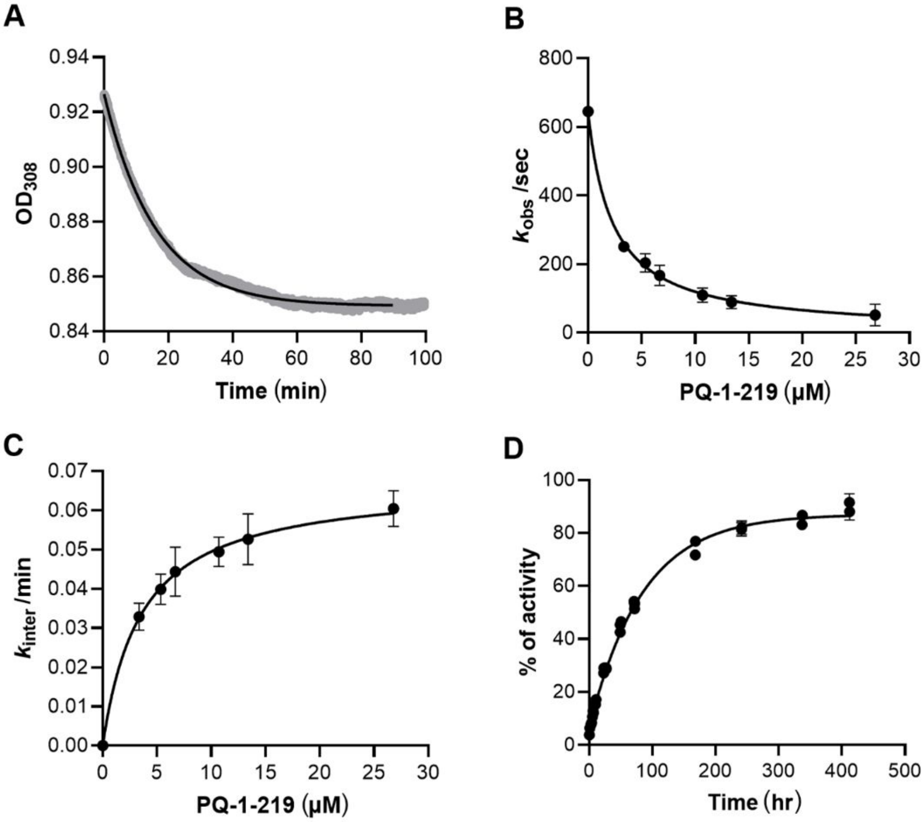

Figure S3. Kinetics of OXA-23 with JDB/PQ-1-219. (A) Representative progress curve of the reaction of OXA-23 with JDB/PQ-1-219 under steady-state condition (10 µM OXA-23 with 100 µM JDB/PQ-1-219). **(B)** The *K*i value of JDB/PQ-1-219 was calculated from the initial velocities of the reaction in the presence of increasing concentration of the inhibitor using nitrocefin as a reporter substrate. **(C)** The inactivation rate constant (k_{inact}) was calculated from the graph of k_{inter} vs JDB/PQ-1-219 concentration. The k_{inter} values were extracted from the progress curves of the competition experiment using nitrocefin as a reporter. **(D)** The k_3 value was measured with the discontinuous jump dilution assay by monitoring the recovery of enzyme activity by time.

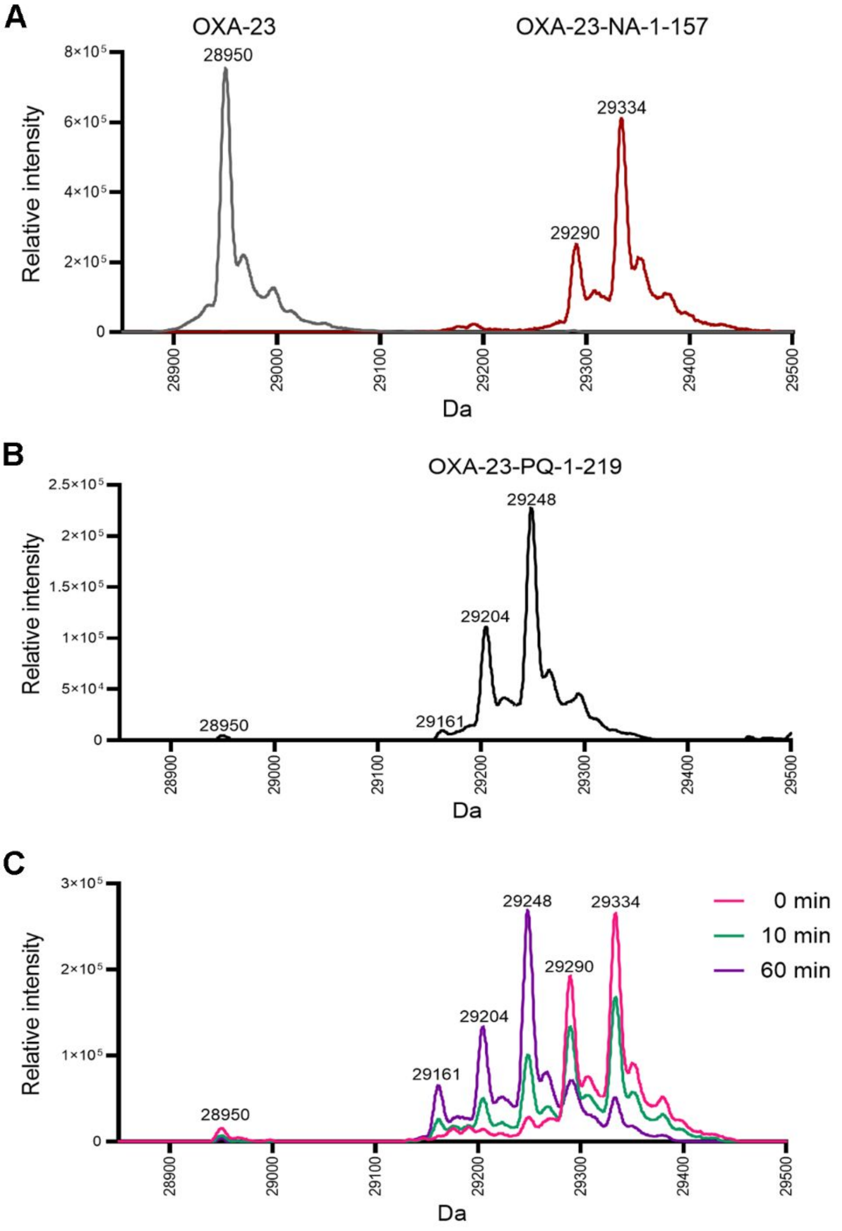

Figure S4. Evaluation of OXA-23-JDB/PQ-1-219 complexes by mass spectrometry. (A) Mass spectra of substrate-free OXA-23 (gray trace) and its complexes formed immediately after incubation with an excess of NA-1-157 (brown trace). **(B)** Mass spectrum of the OXA-23 complexes formed immediately after incubation with an excess of or JDB/PQ-1-219. The major peak(s) of OXA-23 and its complexes are labelled on the spectra with their masses in Da. **(C)** Mass spectra of the OXA-23 complexes formed after 0 s (pink), 10min (green), and 60min (purple) of reaction with 10-fold excess of JDB/PQ-1-219, followed by chasing with an excess of NA-1-157.

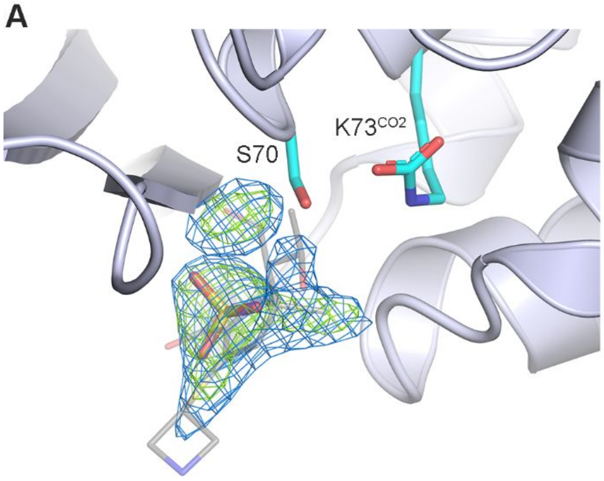

Figure S5. Early time points in the formation of the OXA-23-JDB/PQ-1-219 complex. (A) F_o-F_c electron density (green mesh, 3σ) and Polder difference electron density (blue mesh, 3.5σ) for the 3 min OXA-23-JDB/PQ-1-219 complex. The location of the sulfate is indicated. **(B)** Polder difference electron density (blue mesh, 3.5σ) for the 5 min OXA-23-JDB/PQ-1-219 complex.

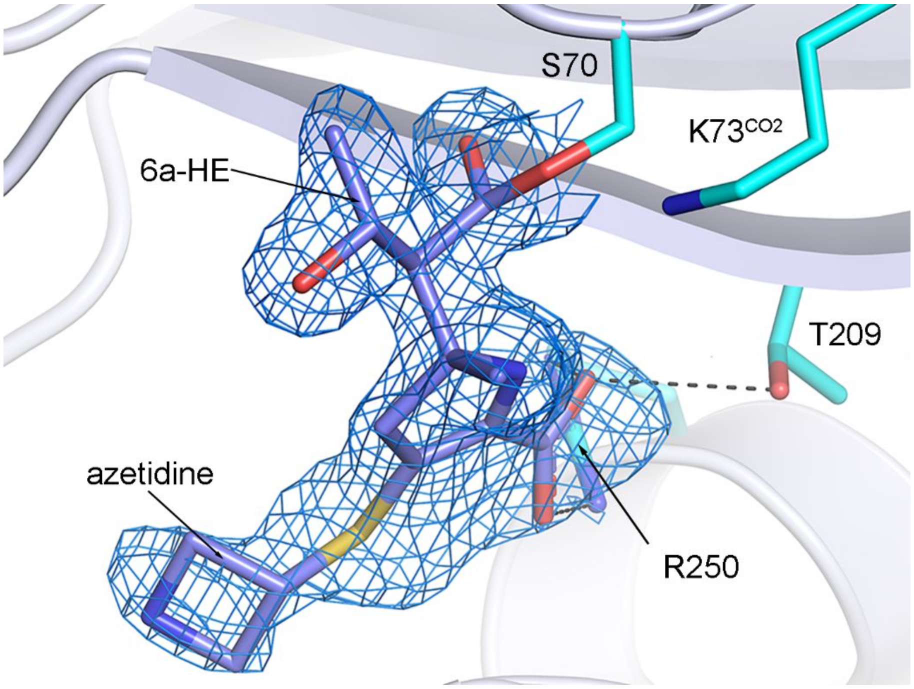

Figure S6. The OXA-23-JDB/PQ-1-219 acyl-enzyme complex. Final 2F_o-F_c density (blue mesh, 1 σ) for the 60 min OXA-23-JDB/PQ-1-219 complex. The 6α-HE group adopts the outward-facing type-II rotamer, and well-ordered density is present for the azetidine tail.

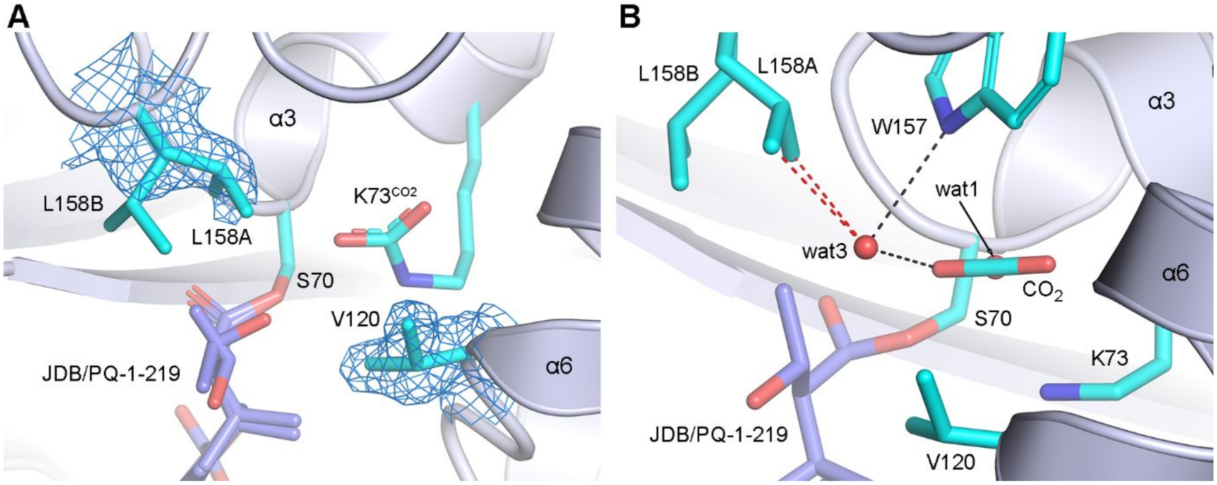

Figure S7. The Leu158 side chain. (A) The 20 min time point, where evidence from the 2F_o-F_c map (blue mesh, 1 σ) shows the presence of two alternate conformations of the Leu158 side chain (Leu158A and Leu158B). **(B)** The 60 min OXA-23-JDB/PQ-1-219 complex, showing the partially-occupied water molecule (wat3) between the free CO_2 and the Leu158A side chain.

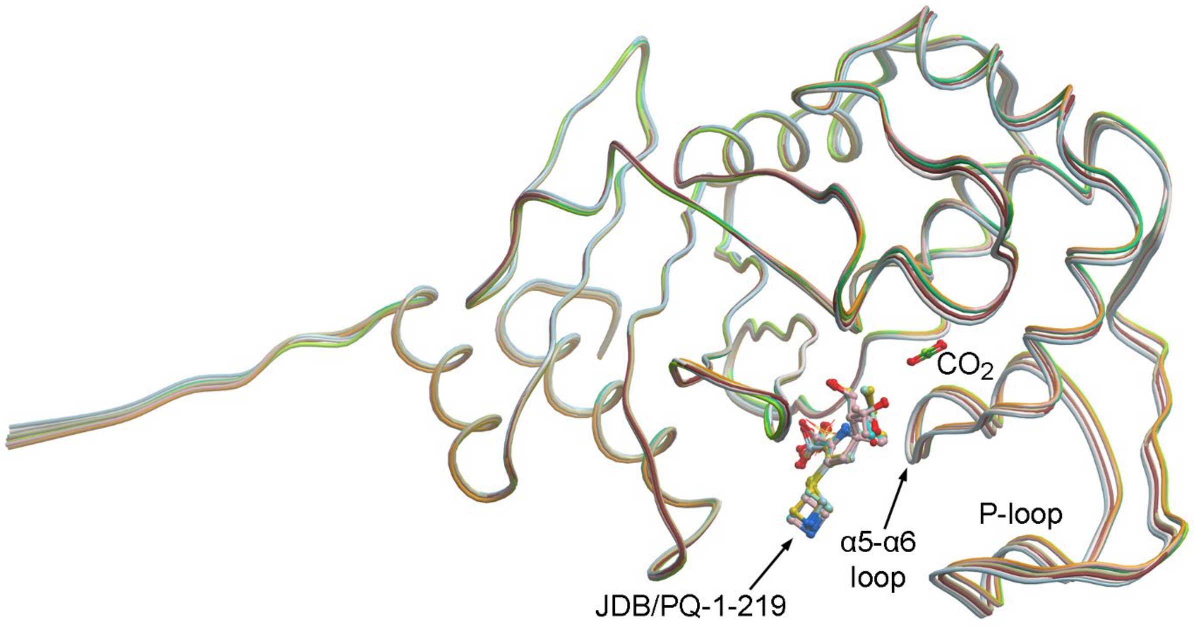

Figure S8. Structure alignment. Superposition of the eight OXA-23-JDB/PQ-1-219 complexes onto substrate-free OXA-23 (PDB code 9NSW) based upon the catalytic sequence (PASTFK) and strand β5. The progressive outward movement of the α5-α6 and P-loop can be seen. Figure drawn with ICM-Pro v. 3.9-3a.[3]

Table S1. Data collection and refinement statistics for the OXA-23-JDB/PQ-1-219 complexes[a].

	3 min	5 min	10 min
Data Collection			
Unit cell, a, b, c (Å)	83.04, 83.04, 84.15	82.98, 82.98, 84.57	83.42, 83.42, 85.22
Resolution (Å)	37.53-1.9 (1.94-1.9)	37.7-1.65 (1.68-1.65)	38.0-1.65 (1.68-1.65)
Reflections - observed	303529	459361	708441
- unique	23825 (1497)	36201 (1760)	36821 (1841)
R_{meas}[b]	0.181 (1.93)	0.089 (0.741)	0.103 (1.166)
R_{pim}[c]	0.050 (0.572)	0.025 (0.213)	0.023 (0.266)
I / σ_I	9.2 (1.5)	13.5 (1.5)	14.2 (1.5)
Completeness (%)	99.9 (99.9)	100 (100)	99.9 (98.9)
CC½[d]	0.996 (0.609)	0.998 (0.907)	0.999 (0.843)
Average multiplicity	12.7 (11.0)	12.7 (11.8)	19.2 (18.2)
Wilson B (Å^2)	27.6	26.2	26.8
Refinement			
PDB Code	9ZOP	9ZOQ	9ZOR
R_{work} / R_{free}[e]	0.2087 / 0.2523	0.2134 / 0.2337	0.1792 / 0.1998
Reflections, work/free	23747 / 1182	36151 / 1761	36766 / 1791
Number of atoms - protein	1992	1992	1984
- water	96	127	136
- ligands	-	-	40[f]
B-factors (Å^2) - protein	31.5	28.5	30.6
- water	36.0	35.6	37.9
- ligands	-	-	38.4
*rmsd*s - bond lengths (Å)	0.009	0.009	0.006
- bond angles (°)	1.17	1.17	1.48
Ramachandran plot[g] - favored (%)	98.4	98.3	97.9
- outliers	0	0	0
Molprobity Score[g]	0.79 (100th percentile)	0.74 (100th percentile)	0.98 (100th percentile)
Molprobity Clashscore[g]	0.99 (100th percentile)	0.74 (99th percentile)	1.96 (99th percentile)

[a] Numbers in parentheses refer to the highest resolution shell.
[b] R_{meas} is the redundancy-independent merging R factor.[4]
[c] R_{pim} is the precision-indicating merging R factor.[4]
[d] Correlation between intensities from random half-sets of data.[5]
[e] R_{free} was calculated using a test set comprising 5% of the data.
[f] JDB/PQ-1-219 was refined as two alternate conformers.
[g] Calculated with the program MOLPROBITY.[6]

Table S1 cont. Data collection and refinement statistics for the OXA-23-JDB/PQ-1-219 complexes[a].

	20 min	30 min	40 min
Data Collection			
Unit cell, a, b, c (Å)	82.64, 82.64, 85.17	82.81, 82.81, 86.29	82.62, 82.62, 86.11
Resolution (Å)	59.3-1.59 (1.62-1.59)	38.3-1.75 (1.78-1.75)	38.2-1.50 (1.53-1.50)
Reflections - observed	778479	601889	935668
- unique	40256 (1881)	30951 (1667)	48334 (2331)
R_{meas}[b]	0.065 (0.707)	0.090 (2.043)	0.057 (1.626)
R_{pim}[c]	0.020 (0.238)	0.020 (0.466)	0.013 (0.374)
I / σ_I	22.4 (2.4)	19.8 (1.7)	24.9 (1.8)
Completeness (%)	99.8 (96.9)	100 (100)	100 (99.6)
CC½[d]	1.0 (0.928)	1.0 (0.825)	1.0 (0.874)
Average multiplicity	19.3 (15.9)	19.4 (18.9)	19.4 (18.5)
Wilson B (Å^2)	24.4	28.0	25.4
Refinement			
PDB Code	9ZOS	9ZOT	9ZOU
R_{work} / R_{free}[e]	0.1940 / 0.2288	0.1804 / 0.1981	0.2061 / 0.2377
Reflections, work/free	39457 / 1972	30878 / 1565	48251 / 2412
Number of atoms - protein	1986	1980	1985
- water	123	97	90
- ligands	40[f]	20	20
B-factors (Å^2) - protein	29.0	32.5	28.5
- water	34.5	36.3	33.0
- ligands	31.2	35.1	32.3
*rmsd*s - bond lengths (Å)	0.005	0.007	0.006
- bond angles (°)	1.46	1.21	1.19
Ramachandran plot[g] - favored (%)	98.8	98.4	98.8
- outliers	0	0	0
Molprobity Score[g]	1.03 (100th percentile)	1.00 (100th percentile)	1.2 (97th percentile)
Molprobity Clashscore[g]	2.45 (99th percentile)	2.24 (99th percentile)	2.22 (99th percentile)

[a] Numbers in parentheses refer to the highest resolution shell.
[b] R_{meas} is the redundancy-independent merging R factor.[4]
[c] R_{pim} is the precision-indicating merging R factor.[4]
[d] Correlation between intensities from random half-sets of data.[5]
[e] R_{free} was calculated using a test set comprising 5% of the data.
[f] JDB/PQ-1-219 was refined as two alternate conformers.
[g] Calculated with the program MOLPROBITY.[6]

Table S1 cont. Data collection and refinement statistics for the OXA-23-JDB/PQ-1-219 complexes[a].

	60 min	90 min
Data Collection		
Unit cell, a, b, c (Å)	82.60, 82.60, 86.78	82.47, 82.47, 84.43
Resolution (Å)	38.4-1.50 (1.52-1.50)	59.8-1.70 (1.73-1.70)
Reflections - observed	691232	444883
- unique	48705 (2276)	33606 (1644)
R_{meas}[b]	0.054 (1.512)	0.126 (0.546)
R_{pim}[c]	0.014 (0.399)	0.047 (0.214)
I / σ_I	20.2 (1.6)	9.7 (1.3)
Completeness (%)	99.9 (97.1)	99.9 (99.0)
CC½[d]	0.999 (0.791)	0.999 (0.984)
Average multiplicity	14.2 (13.8)	13.2 (11.8)
Wilson B (Å^2)	26.3	24.8
Refinement		
PDB Code	9ZOV	9ZOW
R_{work} / R_{free}[e]	0.2009 / 0.2271	0.2015 / 0.2296
Reflections, work/free	46211 / 2435	31798 / 1808
Number of atoms - protein	2017	2011
- water	137	134
- ligands	20	20
B-factors (Å^2) - protein	29.7	27.1
- water	329.2	35.1
- ligands	35.1	33.3
*rmsd*s - bond lengths (Å)	0.002	0.006
- bond angles (°)	1.23	1.16
Ramachandran plot[f] - favored (%)	98.3	98.8
- outliers	0	0
Molprobity Score[f]	0.79 (100th percentile)	0.67 (100th percentile)
Molprobity Clashscore[f]	0.97 (99th percentile)	0.49 (100th percentile)

[a] Numbers in parentheses refer to the highest resolution shell.
[b] R_{meas} is the redundancy-independent merging R factor.[4]
[c] R_{pim} is the precision-indicating merging R factor.[4]
[d] Correlation between intensities from random half-sets of data.[5]
[e] R_{free} was calculated using a test set comprising 5% of the data.
[f] Calculated with the program MOLPROBITY.[6]

SUPPLEMENTAL REFERENCES

1. Ueda, Y.; Roberge, G. Carbapenem intermediates. GB2173801A, 1986.
2. Nagao, Y.; Abe, T.; Shimizu, H.; Kumagai, T.; Inoue, Y., Asymmetric synthesis of new non-natural 1β-methylcarbapenems bearing methylthio group at the C6 position. *Heterocycles* **1992,** *33*, 523-528.
3. Abagyan, R. A.; Totrov, M. M.; Kuznetsov, D. A., ICM: A new method for protein modeling and design: Applications to docking and structure prediction from the distorted native conformation. *J. Comp. Chem.* **1994,** *15*, 488-506.
4. Weiss, M. S., Glocal indicators of X-ray data quality. *J. Appl. Cryst.* **2001,** *34*, 130-135.
5. Karplus, P. A.; Diederichs, K., Linking crystallographic model and data quality. *Science* **2012,** *336*, 1030-1033.
6. Chen, V. B.; Arendall, W. B.; Headd, J. J.; Keedy, D. A.; Immormino, R. M.; Kapral, G. J.; Murray, L. W.; Richardson, J. S.; Richardson, D. C., MolProbity: All-atom structure validation for macromolecular crystallography. *Acta Crystallogr.* **2010,** *D66*, 12-21.
